# Super-Resolution Imaging of Nuclear Pore Responses to Mechanical Stress and Energy Depletion

**DOI:** 10.3390/v18020167

**Published:** 2026-01-27

**Authors:** Dariana Torres-Rivera, Sobhan Haghparast, Bernd Rieger, Gregory B. Melikyan

**Affiliations:** 1 Department of Pediatrics, Emory University, Atlanta, GA 30322, USA; dariana.torres.rivera@emory.edu; 2 Faculty of Applied Sciences, Delft University of Technology, 2628 Delft, The Netherlands; s.haghparast@tudelft.nl (S.H.); b.rieger@tudelft.nl (B.R.); 3 Children’s Healthcare of Atlanta, Atlanta, GA 30329, USA

**Keywords:** nuclear pore complex, single-molecule localization microscopy, human immunodeficiency virus, osmotic stress, ATP depletion

## Abstract

HIV-1 entry into host cells culminates in integration of the reverse transcribed double-stranded viral DNA into host genes. Several lines of evidence suggest that intact, or nearly intact, HIV-1 cores—large, ~60 nm-wide structures—pass through the nuclear pore complex (NPC), and that this passage is associated with pore remodeling. Cryo-electron tomography studies support the dynamic nature of NPCs and their regulation by cytoskeleton and ATP-dependent processes. To explore NPC remodeling, we used super-resolution Stochastic Optical Reconstruction Microscopy (STORM) of U2OS cells endogenously expressing nucleoporin 96 tagged with SNAP. Single-molecule localization imaging and computational averaging resolved 8-fold symmetric nuclear pores with an average radius of ~51 nm. Depletion of cellular ATP using sodium azide or antimycin A, previously reported to reduce the size of yeast NPCs, did not significantly alter the nuclear pore radius in U2OS cells. Similarly, stressing the nuclear envelope by hypotonic or hypertonic conditions failed to induce detectable expansion or contraction of NPCs. These results indicate that the NPCs in U2OS cells do not respond to ATP depletion nor mechanical stresses on changes in pore morphology that can be resolved by STORM. Since these cells are infectable by HIV-1, we surmise that direct multivalent interactions between HIV-1 capsid and phenylalanine-glycine nucleoporins lining the pore’s interior drive the core penetration into the nucleus and the associated changes in the pore structure.

## 1. Introduction

The nuclear pore complex (NPC) is a ~120 MDa multiprotein complex that spans the nuclear envelope (NE) and controls the transport of multiple proteins in and out of the nucleus [1,2,3,4]. The NPC is composed of nucleoporins (NUPs), which are classified as scaffold NUPs and phenylalanine-glycine-NUPs (FG-NUPs). The scaffold NUPs are structural proteins that form the pore and are directly attached to the NE [2,3,4,5]. The Y-complex is a part of the scaffold and consists of NUP96, NUP107, and NUP133, among other NUPs [2,5]. The FG-NUPs are flexible NUPs that contain FG repeats, which generates a liquid-phase separation inside the inner ring of the pore that serves as a selective filter for transport of host factors [2,6,7].

The NPC has been previously considered a static structure that allowed the passage of molecules in/out of the nucleus in a size-dependent manner. Small molecules permeate the pore through passive diffusion, while larger molecules require active transport to enter in the nucleus, which is mediated by transport factors like Importin β [2,3,8,9]. Passive diffusion through the NPC is generally limited for molecules larger than 60 kDa [2,3]. Furthermore, CryoEM imaging of isolated nuclei suggested that the diameter of the central pore was ~40 nm [5]. However, recent evidence using Cryo-FIB milling and imaging native, *in cellulo* pore structures has revealed that NPCs of mammalian cells have larger inner pore diameter, close to 64 nm [10,11,12]. In addition, Zimmerli et al. [13] have shown that yeast NPCs are dynamic, and that ATP is required to maintain an expanded state of the pore. In addition, this study and a recent work using expansion fluorescence microscopy have also found that hyperosmotic shock reduces the NPC size [13,14], suggesting that the nuclear envelope tension can modulate the pore size. It is worth noting that the pore size can be heterogeneous within the same cell, and that NPCs located at the bottom and top of the NE have been reported to have different diameters [14]. Neuronal cell differentiation and *Xenopus* oocyte development can also promote changes in the NPC architecture [15,16,17]. All these findings support the notion that the NPC is a dynamic structure that may, in principle, allow the passage of very large macromolecular complexes.

Perhaps one of the largest cargoes that can pass through the NPC is the cone-shaped HIV-1 capsid core, which measures ~60 nm at the widest end [10,18]. The presence of cone-shaped HIV-1 cores inside the nucleus of infected cells supports the ability of intact or nearly intact capsid cores to penetrate the NPCs [10,18,19,20,21,22,23]. Interestingly, a fraction of NPCs appeared deformed or cracked in the vicinity of HIV-1 capsids inside the nucleus [18,19,24]. These studies support a flexible model for the NPC that can facilitate the passage of very large macromolecular complexes, like the HIV-1 capsid core.

Based on the above findings, we hypothesize that the nuclear pore may respond to mechanical stimuli and/or require ATP to sustain an enlarged state. To explore the NPC dynamics, we used U2OS cells endogenously expressing NUP96-SNAP and performed Single Molecule Localization (SML) STORM microscopy to visualize the NPC architecture. We first confirmed that U2OS NUP96-SNAP cells are readily infectable by VSV-G pseudotyped HIV-1, and that the nuclear import of viral pre-integration complexes is not delayed compared to parental U2OS cells. Depletion of ATP with sodium azide or Antimycin A did not significantly affect the NPC radius. Similarly, mechanically stressing the nuclear envelope by osmotic swelling or by hyperosmotic shock did not cause a detectable increase or decrease of the NPC diameter, respectively. Taken together, nuclear pore size in U2OS cells appears to be tolerant to both ATP depletion and mechanical stresses. Comparing our data with previous studies reporting changes in NPC morphology [10,13,19,25,26], we surmise that the limited spatial resolution of SML microscopy (around 13 nm) and NPC heterogeneity may mask the potential effects of NPC stretching and energy depletion on their size. Additionally, differences in cell types or their physiological state can contribute to NPC’s responses to mechanical stimuli.

## 2. Methods

### 2.1. Cells and Reagents

U2OS was obtained from Philip Santangelo (Georgia Institute of Technology, Atlanta, GA, USA), HEK293T/17 cells from ATCC (#CRL11268, Manassas, VA, USA), and TZM-bl cells from the BEI Resources (NIAID, NIH; HRP-8129, Manassas, VA, USA). U2OS NUP96-SNAP clone #33, in which SNAP was knocked-in at the C-terminus of NUP96 using CRISPR/Cas9 technology, was obtained from Cell Lines Services (CLS GmbH #300444, Eppelheim, Germany; see Thevathasan et al., 2019 [27]). All cells were maintained in Dulbecco’s modified Eagle’s medium (DMEM, Corning, New York, NY, USA, #10-013-CV), supplemented with 10% Fetal Bovine Serum (FBS, Hyclone, #SV30014.03, Logan, UT, USA) and 100 U/mL of Penicillin/Streptomycin (P/S, Gemini Bio-products #400-109, West Sacramento, CA, USA) at 37 °C, 5% CO_2_. HEPES-based live cell imaging buffer was obtained from Invitrogen (#A14291DJ, Waltham, MA, USA). Sorbitol (PHR1006) and reagents used for ATP depletion, 2-deoxy-D-glucose (2-dDG, #D8375-1G), antimycin A (Ant-A, #A8674), and sodium azide (NaN_3_, #S8032) were obtained from Sigma (St. Louis, MO, USA).

### 2.2. Production and Characterization of HIV-1 Pseudoviruses

To produce luciferase reporter viruses, HIV-1 pseudoviruses were made by transfecting HEK293T/17 cells with pVSV-G (0.5 µg, a gift from Mamuka Kvaratskhelia, U. Denver) and pNL4-3 R-E Luciferase (2 µg, from Anna Cereseto, University of Trento, Trento, Italy). For producing fluorescent viruses, cells were transfected with pVSV-G (0.5 µg), NL4-3 eGFP (2 µg, from Chris Aiken, Vanderbilt, Nashville, TN, USA), and Integrase-superfolder GFP (pVpr-PC-IN-sfGFP, 0.8 µg; from Anna Cereseto). Transfection was done using JetPrime reagent (PolyPlus transfection, #114-15), according to the manufacturer’s instructions. Briefly, plasmids were mixed at 1:2 DNA:JetPrime ratio in 200 µL of JETPrime Buffer and added to cells. Transfection medium was changed to complete DMEM medium without Phenol Red (Gibco, ThermoFisher, #31053-028, Baltimore, MD, USA) at 6 h after transfection, and viruses were collected after 48 h. Cell supernatant was collected and filtered with 0.45 µm of polyethersulfone Nalgene syringe filters (ThermoFisher, #725-2545, Waltham, MA, USA), aliquoted and stored at −80 °C. Viruses were characterized by measuring reverse transcriptase activity (RT) using qPCR [28]. This method measures RT activity as a reference for mature virions.

For PF74 time-of-addition assay, 1 × 10^4^ cells/well of TZM-bl, U2OS, and U2OS NUP96-SNAP cells were seeded on a 96-well black clear-bottom plate (Corning #3603) and cultured overnight. NL4-3 Luciferase-expressing pseudovirus in complete DMEM medium were added to cells (0.8 RTU/mL) and spinoculated at 1550× *g* for 30 min, 4 °C. Medium was changed to complete DMEM, and cells were incubated at 37 °C. Two µM of PF74 (PF-3450074, Sigma #SML0835, St. Louis, MO, USA) were added at indicated times post-infection (with a 2 h interval) up to 24 h.p.i. Luciferase activity was measured at 48 h.p.i. by mixing 1:1 Luciferase reagent Bright Glow (Promega #2620, Wisconsin, IL, USA) with 1X Glo Lysis Buffer (Promega #E2661, Madison, WI, USA). Luciferase signal was measured on a PerkinElmer TopCount NXT (Shelton, CT, USA) luminescence plate reader.

To measure nuclear import of fluorescently labeled viruses, TZM-bl, U2OS, and U2OS NUP96-SNAP cells were seeded on collagen-coated 8-well coverslips (Nunc Brand #177402, ThermoFisher, Waltham, MA, USA) at 4 × 10^4^ cells/well and cultured overnight. Cells were then infected with NL4-3eGFP INsfGFP viruses (0.65 RTU/mL) in complete DMEM by spinoculation at 1550× *g* for 30 min, 4 °C. Medium was changed with fresh complete DMEM, and cells were incubated at 37 °C for 4 h. Cells were fixed, immunostained for Lamin with Rabbit Anti-Lamin B1 (AbCam #16048, Cambridge, UK) and secondary AlexaFluor 647 Goat anti-rabbit IgG (H + L) (ThermoFisher #A21245), and imaged on a Zeiss LSM880 confocal microscope (Carl Zeiss Group, Oberkochen, Germany).

### 2.3. ATP Depletion

For ATP depletion experiments, 50 µL of 5 × 10^5^/mL U2OS NUP96-SNAP cells were seeded in 96-well black clear-bottom plates and cultured overnight. Next day, cells were washed with Phosphate Buffer Saline without calcium and magnesium (PBS^−/−^, Corning #21-040-CV) and incubated with complete medium (DMEM, 10% FBS and 1% P/S, abbreviated (+)Gluc), glucose-free medium (Live cell imaging buffer, 10% FBS and 1% P/S, abbreviated (−)Gluc), or ATP depletion medium for different times. ATP depletion medium was made by supplementing (−)Gluc buffer with 10 mM of NaN_3_ and 6 mM of 2-dDG, or with 10 µM of Antimycin A and 20 mM of 2-dDG. Cell’s ATP levels were determined using the PerkinElmer ATPlite assay kit (#6016943), following manufacturer’s instructions. Briefly, ATP standards were resuspended and serially diluted 1:10. Cells were then lysed with lysis solution from the kit and shaken for 5 min at 7000 rpm. After lysis, substrate solution was added to cells and shaken for an additional 5 min at 7000 rpm. Lysates were then incubated for 10 min in dark. The resulting luciferase activity was measured using the PerkinElmer TopCount NXT luminescence plate reader.

### 2.4. Osmotic Stretching and Shrinkage of Nuclear Envelope

For osmotic stretching of NE, U2OS NUP96-SNAP cells were seeded in 35 mm collagen-coated dishes (Corning # 430165) and cultured overnight. Cells were then transiently transfected with 0.2 µg of pSB-CMV-MCS-Puro-cPLA2-EGFP (Addgene #162568, Watertown, MA, USA) with 1:2 DNA:JETPrime reagent and mixed in 200 µL of JETPrime Buffer. After 6 h, transfection medium was replaced with complete DMEM medium supplemented with 3 µg/mL aphidicolin (Millipore-Sigma #178273-1MG, Burlington, VT, USA), to prevent cell division, and cultured overnight at 37 °C. To promote nuclear envelope swelling, cells were treated according to Shen et al. [29] and Enyedi et al. [30]. Briefly, cells were permeabilized for 5 min at 37 °C with 25 µg/mL of digitonin (#43065-0.1; Research Product International (RPI); Mount Prospect, IL, USA) in the presence of 5% Polyvinylpyrrolidone (PVP360, Sigma #9003-39-8) dissolved in base medium (BM; 123 mM of KCl, 12 mM of NaCl, 1.94 mM of MgCl_2_, 0.28 mM of CaCl_2_, 1 mM of EGTA pH 7.2 (Sigma #E3889-25G), 10 mM of HEPES (ThermoFisher Scientific #15630080)). Next, medium was changed to BM containing 5% or 0% PVP360 and incubated for 5 min or 15 min at 37 °C, respectively. Cells were then fixed with 4% Paraformaldehyde (PFA, Electron Microscopy Sciences #15710, Hatfield, PA, USA) for 10 min, and PFA activity was quenched with 20 mM Tris pH 8.0 (G-Biosciences #R002, St. Louis, MO, USA) for 5 min at room temperature. Samples were blocked with 20% FBS for 30 min and stained with 0.5 µM SNAP-Surface AF647 (New England Biolabs #S9136S, Ipswich, MA, USA) with 1 µM of dithiothreitol (DTT, American Bioanalytical #AB00490, Natick, MA, USA) for 1 h at room temperature.

For shrinkage of NE, U2OS NUP96-SNAP cells were seeded in collagen coated 8-well chamber coverslips with 3 µg/mL of aphidicolin incubated overnight at 37 °C. Cells were then cultured with 2 M of sorbitol (hyperosmotic condition) for 1 h at 37 °C. Cells were fixed with 2% PFA in the presence of 500 mM of sorbitol for 1 min, permeabilized with 0.1% digitonin also in the presence of 500 mM of sorbitol for 30 min and fixed again with 2% PFA for 10 min at room temperature. Fixation was quenched with 20 mM of Tris pH 8.0 for 5 min. Cells were then blocked with 20% FBS for 30 min and stained with 0.5 µM of SNAP-AF647 supplemented with 1 µM DTT.

### 2.5. Preparation of Samples for Imaging

For ATP depletion experiments, U2OS NUP96-SNAP cells (10^5^ cells/well) were seeded on collagen-coated 8-well chamber coverslips (Nunc Brand #177402) with 3 µg/mL aphidicolin, to prevent cell division, and cultured overnight at 37 °C. Next day, cells were treated with (+)Gluc, (−)Gluc or ATP depletion medium (10 mM of NaN_3_ with 6 mM 2-dDG or 10 mM Antimycin A with 20 mM 2-dDG) at indicated times. For Supplementary Figure S6, Antimycin A treated samples were co-treated with 2 µM nocodazole (Noco, Honeywell Fluka, Seelze, Germany), or left untreated, for 4 h. All samples were then pre-fixed with 2% PFA for 30 s, permeabilized with 0.1% digitonin for 30 min, and post-fixed with 2% PFA for 10 min. PFA was quenched with 20 mM of Tris pH 8.0 for 5 min. Samples were blocked with 10–20% FBS for 30 min, stained with 0.5–1 µM SNAP-AF647 with 1 µM DTT for 1 h at room temperature, and washed with 10% FBS.

For STORM imaging, a STORM buffer was prepared based on Nikon’s N-STORM buffer recommendations [31]. Briefly, Buffer A contained 10 mM of Tris pH 8.0 and 50 mM of NaCl, and Buffer B contained 50 mM of Tris pH 8.0, 10 mM of NaCl, and 10% glucose (D-Glucose anhydrous; Gibco #15023-021) in PBS+/+. GLOX solution was prepared by adding 14 mg of Glucose Oxidase (Sigma #G2133-50KU) and 50 µL of catalase (17 mg/mL; Sigma #C40-500MG) into 200 µL of Buffer A. Finally, 50–100 mM MEA (Cysteamine Hydrochloride; Sigma #M6500-25G) and 1% GLOX solution were mixed with Buffer B and added to samples. The wells were then sealed with parafilm and imaged immediately (see below). STORM buffer was replaced every 2 h.

### 2.6. Confocal and STORM Imaging

Confocal images were taken on a Zeiss LSM880 confocal microscope using a 63×, 1.4NA oil immersion objective. The image dimensions were 512 × 512 pixels, with zoom of 2 (pixel size of 0.132 µm). The pixel dwell time was 2.05 µs, and 2-line averaging was used to reduce background noise. Z-stacking was performed with 0.5 µm interval and a pinhole of 100 µm. Images were taken using the filter sets 488/561/647 nm. SNAP-AF647 was excited with 647 nm laser, and cPLA2-GFP was excited with 488 nm laser.

For STORM images, we used an Oxford Nanoimager (ONi, Oxford, England) SML microscope equipped with an Olympus 100×, 1.4NA oil immersion objective, and a 640 nm dichroic mirror. The excitation power density was ~4 kW/cm^2^ for 488/561/647 nm lasers (0.24 kW/cm^2^ for 405 nm UV laser). Before imaging, a 2-channel color mapping calibration was performed using 0.1 µm TetraSpeck microspheres (Invitrogen #T7279) emitting in blue, green, orange, and far red. A mapping calibration with less than 20 nm of Standard Deviation of Errors was accepted. After calibration, drift correction was tested by imaging coverslip-adhered TetraSpeck beads with 60 ms exposure time (16 frames per second) using 640 nm and 561 nm lasers, with 100 frames each and with a 45° (semiTIRF) illumination angle. Calibration/drift correction was confirmed by visual inspection of both colors colocalizing. After calibration, cells were imaged using 60 ms exposure time in a semiTIRF mode, and frames were acquired as follows: (1) 10–40 frames of 640 nm laser excitation with 1.7 mW of laser power; (2) pre-bleaching of SNAP-AF647 with 1500 frames at the highest power (~180 mW) of 640 nm laser; (3) blinking of SNAP-AF647 started at 120 mW of 640 nm laser and imaged for 5000 frames. Blinking was then enhanced with exposure of 0.2 mW of 405 nm laser in conjunction with 120 mW of 640 nm laser for an additional 5000 frames.

### 2.7. Image Analysis and Statistics

Confocal images were processed with Zeiss ZEN (black edition) version 8.0.0.273 software and ImageJ version 2.3.0. Normalized gray values of nuclear envelope fluorescence were obtained through line histogram plugin on ImageJ. The volume of nucleus was calculated using the HKMeans plugin on ICY software version 2.5.4.0 (https://icy.bioimageanalysis.org/, accessed on 24 January 2026). Half times for HIV-1 nuclear import (T_1/2_) from PF74 addition were determined by fitting with exponential function using GraphPad Prism 10.3.1. Fluorescently labeled HIV-1 INsfGFP particles inside the nucleus were counted manually. Statistical analyses for ATP depletion luciferase assays, nuclear envelope volumes, and NPC radius were done using GraphPad. Brown-Forsythe and Welch ANOVA were used for all statistical analyses unless stated otherwise.

For visualizing STORM images, drift correction, filtering, and exporting of localization coordinates, we used NimOS version 1.19.1 software and the cloud-based CODI (https://alto.codi.bio, accessed on 24 January 2026) program developed by ONi. Drift correction was performed using the built-in drift correction feature in NimOS. Localizations were filtered by removing localizations with less than 500 photons and with more than 5000 photons. In addition, events with localization precision worse than 20 nm were discarded. Averaging of NPCs were performed using a MATLAB 2024b script developed by the Rieger lab (Delft, The Netherlands) [32,33]. For this analysis, each NPC exhibiting symmetric circular appearance was manually picked from the field of view by cropping the point cloud with 180 × 180 nm boxes. For each dataset, all NPCs were initially centered by subtracting their mean localization coordinate from all coordinates and the cartesian coordinates were then transformed to polar coordinates ($\left(x,y\right)\to (\rho ,\theta$)). To generate images of averaged NPCs, we applied the template-free registration-based particle averaging method developed by Wang et al. [33]. Briefly, individual NPC localizations were iteratively aligned using pairwise registration based on structural similarity. This process is corrected for translational and rotational variations across particles. The aligned NPCs were then combined to produce a high signal-to-noise composite image that emphasizes consistent structure of NPCs. The FRC curve was calculated, as previously established [34]. Briefly, the dataset was divided into two halves, and each half was registered, followed by the application of eight-fold symmetry to each particle. This ensures alignment accuracy and minimizes bias while enhancing the signal-to-noise ratio. After applying the rotational symmetry, the FRC curve was computed and plotted. By applying a resolution threshold to the FRC curve, the resolution of the reconstruction was determined, providing a quantitative assessment of the reconstructed structure.

The mean radius of the pores was computed as follows. First, the localizations were centered by subtracting their mean coordinates and by transforming the localizations from Cartesian to polar coordinates, as mentioned previously. The localizations were superimposed to create a 2D histogram of the NPC SMLs. The radial distance from center of the localizations were plotted, fitted by a Gaussian distribution and normalized to the number of localizations. Lastly, the mean *µ* and standard deviation *σ* were calculated from the Gaussian fit. The *µ* position of the Gaussian does not showcase the correct average of the pore size, so the peak position was corrected to first order by $-\frac{1}{2}\;{\left(\frac{\sigma }{\mu }\right)}^{2}$ [17,35]. A total of 100,000–400,000 SMLs from several hundreds to several thousands of NPCs were used to create the superimposed NPCs and to calculate the mean radius from the Gaussian fit after removing outliers (SMLs farther than 100 nm from center and closer than 10 nm from center).

## 3. Results

### 3.1. NUP96-SNAP Tag Does Not Affect the HIV-1 Nuclear Import Kinetics

We chose to visualize the NPC complexes by using tagged NUP96, 32 copies of which are present in the nuclear pore complex [36]. We employed the U2OS cell line in which the endogenous locus of NUP96 was tagged with SNAP using a CRISPR/Cas9 knock-in approach (described in Thevathasan et al. [27]). These cells were previously used to visualize the nuclear pore structure by super-resolution microscopy, yielding a pore radius of 53–56 nm [27,37]. We first tested whether the SNAP-tagged NUP96 can affect HIV-1 nuclear import and infectivity. Parental U2OS cells and U2OS NUP96-SNAP cells, as well as control HeLa-derived reporter TZM-bl cells, were infected with NL4-3 pseudoviruses encoding for luciferase. The kinetics of nuclear import was measured by adding 2 µM of PF74, a compound that binds to HIV-1 capsid [38,39], at different times post-infection (p.i.) and reading the resulting luciferase activity at 48 h.p.i. (Supplementary Figure S1A). Since 2 µM of PF74 are known to inhibit HIV-1 nuclear import but not reverse transcription [38,40,41,42], the luciferase signal as a function of time of PF74 addition reports the HIV-1 nuclear import kinetics. The overall efficiency of infection was evaluated at the 24 h time point, at which the signal reached a plateau (Supplementary Figure S1A). The overall efficiency of infection was ~2-fold lower in U2OS NUP96-SNAP compared to parental U2OS cells, and 4.5-fold lower than TZM-bl (Supplementary Figure S1A). However, the kinetics of nuclear import was not delayed by SNAP-tagging of NUP96. The estimated half times (T_1/2_) of nuclear import in U2OS NUP96-SNAP and parental cells was 4.1 h and 5.1 h, respectively. These times were close to T_1/2_ of 5.3 h for TZM-bl cells. To more directly evaluate the efficiency of nuclear import, we measured the number of fluorescently labeled HIV-1 cores inside the nucleus around the half-time of infection (4 h.p.i.) (Supplementary Figure S1B,C). Cells were infected with NL4-3 pseudoviruses labeled with integrase-superfolder GFP (INsfGFP) and counted the number of fluorescent spots per nucleus (Supplementary Figure S1C). Although U2OS NUP96-SNAP cells exhibited fewer HIV-1 cores per nucleus than parental cells, in agreement with their reduced infectivity (Supplementary Figure S1A), the difference did not reach statistical significance. These results suggest that the nuclear import of HIV-1 is not majorly reduced by tagging NUP96. In contrast, the number of nuclear cores in TZM-bl cells was significantly higher compared to both parental U2OS and U2OS NUP96-SNAP cells, highlighting cell type-dependent differences in the efficiency of HIV-1 infection.

### 3.2. ATP Depletion Does Not Cause Detectable Changes in NPC Size

**Figure 1:**
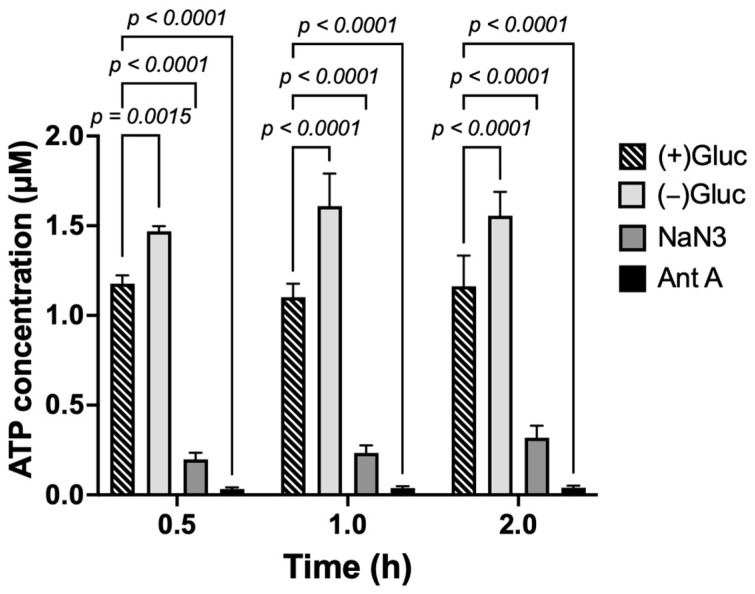
ATP depletion of U2OS NUP96-SNAP cells. Cells were incubated in complete DMEM with 10% FBS ((+)Gluc), in HEPES-based imaging buffer without glucose ((−)Gluc), or in ATP depletion buffer: 10 mM of sodium azide with 6 mM of 2-deoxy-D-glucose (NaN_3_) or 10 µM of Antimycin A with 20 mM of 2-deoxy-D-glucose (Ant-A) at different times. The cellular ATP concentration (in µM) was measured in triplicate samples using a kit and plotted as mean ± SD. Statistical analysis was done with two-way ANOVA. (See Supplementary Figure S2 for an independent replicate).

Since the yeast NPCs have been reported to constrict upon depletion of cellular ATP [13], we asked whether depleting ATP in mammalian cells would have the same effect. Cells were depleted of ATP using two approaches: (1) incubation with 10 mM of sodium azide, in combination with 6 mM 2-deoxy-D-glucose (dubbed NaN_3_), or (2) treatment with 10 µM of Antimycin A with 20 mM 2-deoxy-D-glucose (Ant-A). Our ATP depletion protocols were validated by measuring the ATP concentration in U2OS NUP96-SNAP cells (Figure 1). Cells incubated in HEPES-based imaging buffer without glucose ((−)Gluc) or in complete cell medium ((+)Gluc) were used as controls (see Section 2). Cellular ATP concentrations significantly dropped after NaN_3_ and Ant-A treatment compared to (+)Gluc- or (−)Gluc-treated cells. ATP depletion with Ant-A was more efficient than with NaN_3_. Interestingly, cells treated with (−)Gluc medium contained more ATP than cells treated in (+)Gluc, suggesting that glucose starvation activates alternative metabolic pathways to increase ATP production [43,44,45]. A dramatic depletion of cellular ATP using these protocols was reproducibly observed in multiple replicates, even after 10 min of incubation (Supplementary Figure S2). Also, the ATP levels remained the lowest after 4 h of incubation with Ant-A (Supplementary Figure S2B).

In single-molecule localization microscopy (SMLM), molecules transition between dark and emissive states, which are localized in each time frame and fitted with a Point Spread Function model. The fluorescent events are spatially sparsely distributed. Thus, the position of a molecule can be pinpointed with much higher accuracy than the diffraction limit. From the estimated positions of many thousands of localizations, a super resolution image can be reconstructed [46,47].

**Figure 2:**
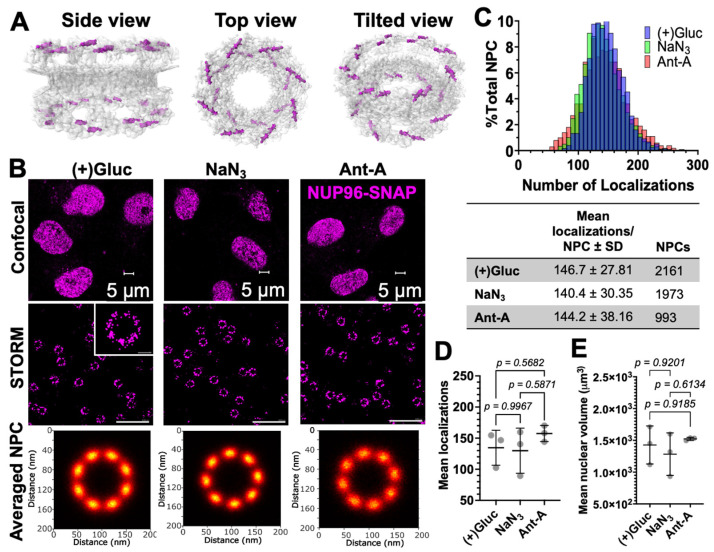
STORM imaging of NPCs on ATP depleted samples. (**A**) EM density (grey) images of the NPC with NUP96 highlighted (magenta) viewed from the side, top, and tilted. Structure was taken from the Protein Data Bank (PDB): 7TBJ. (**B**) *Top*: Confocal images of U2OS NUP96-SNAP cells incubated in complete medium ((+)Gluc) and ATP-depletion media, NaN_3_ or Ant-A, for 30 min at 37 °C. Cells were then fixed with PFA, permeabilized, and NUP96-SNAP was stained with the SNAP-AF647 dye. Shown are representative images of confocal slices at the bottom of the nuclear envelope. *Middle*: STORM images (AF647 localizations) of NPCs at the bottom of the nuclear envelope. Scale bar: 0.5 µm. *Inset*: zoomed in of one NPC (scale bar 50 nm). *Bottom*: Images of averaged NPCs from STORM images. NPCs were selected manually, and averaging was performed using a MATLAB script. (**C**) Distribution of the number of SNAP-AF647 dye localizations per NPC obtained by STORM (panel (**B**)). Table shows mean localizations per NPC ± SD and the number of NPCs analyzed. (**D**) Mean number of localizations for each biological replicate. (See Supplementary Figures S4–S6 for independent replicates). Statistical analysis was done with Brown-Forsythe and Welch ANOVA. (**E**) Mean nuclear volumes (µm^3^) for (+)Gluc, NaN_3_, and Ant-A treated samples. Shown are mean ± SD from three biological replicates. Brown-Forsythe and Welch ANOVA were used for statistical analysis.

Using Stochastic Optical Reconstruction Microscopy (STORM), we next measured the NPC radii in control and ATP depleted cells (Figure 2). Since the average NPC diameter reported for the Y-complex, comprising NUP96, is ~107 nm [27,48], changes in the pore size exceeding 20% (~20 nm) should be resolvable by STORM. The NPC structure with NUP96 highlighted is shown in Figure 2A. Cells were depleted of ATP with NaN_3_ or Ant-A for 30 min, fixed, and stained for NUP96-SNAP with the SNAP-reactive SNAP-AF647 dye. Initial examination by confocal microscopy showed no apparent changes in NE morphology for cells in (+)Gluc vs. ATP depletion buffer (Figure 2B, top). STORM images revealed NPCs with different appearances with varied apparent spoke arrangements, including the expected 8-fold symmetry (Figure 2B, middle, *inset*). The varied pore appearance can be due to incomplete labeling of NUP96-SNAP, which is around 60% for the SNAP-AF647 dye [27], and/or may reflect the overall NPC heterogeneity [25,26]. To measure the NPC size, we manually selected NPCs, from 680 to 2470 NPCs, with a symmetric circular appearance. Next, the selected pores were centered by subtracting their center of mass, the cartesian coordinates were transformed into polar coordinates, and all SMLs were superimposed to form an ensemble of NPC localizations. We also applied a template-free registration-based composite to the averaged NPC pores to visualize the NPC symmetry (Figure 2B, bottom). The spatial resolution determined by Fourier Ring Correlation (FRC) analysis was about 13.6 nm for (+)Gluc-treated samples (Supplementary Figure S3), in excellent agreement with 13.3 nm reported by Thevathasan et al. [27] in SNAP-AF647-labeled NUP96-SNAP U2OS cells. The number of localizations per NPC, and, therefore, the NUP96-SNAP expression level and/or labeling efficiency were not affected by ATP depletion (Figure 2C,D). We also found that the nuclear volume did not change after depleting ATP (Figure 2E).

**Figure 3:**
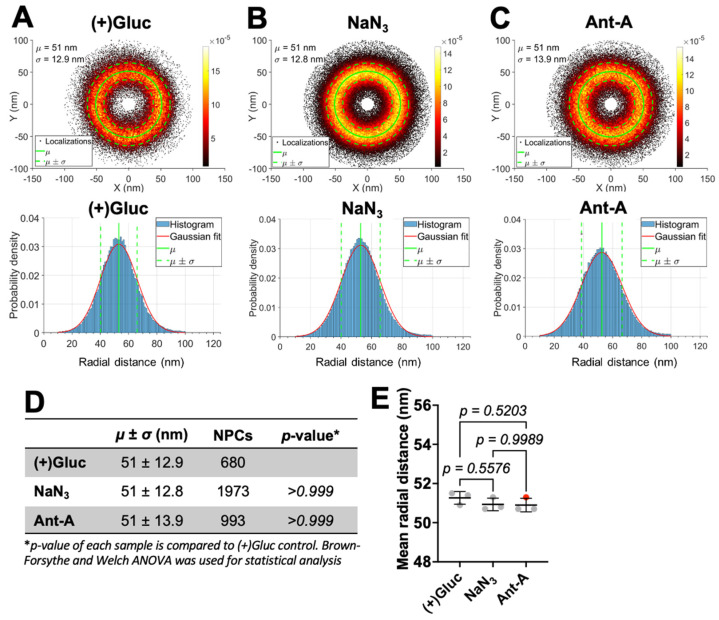
ATP depletion does not change the NPC size. (**A**–**C**) *Top*: Scatter plots showing the localizations of super imposed NPCs analyzed in Figure 2 for (+)Gluc (**A**), NaN_3_ (**B**), and Ant-A (**C**) samples. We aligned the centers of mass for each NPC SMLs, transformed localizations into polar coordinates, and then superimposed to obtain a density map of all localizations for the analyzed pores. Shown right is the pseudo color scale for the localization density. *Bottom*: SML radial distance histograms were fitted with a Gaussian function (red). The mean radius (*µ*) and standard deviation (*σ*) were calculated from the fitted Gaussian distribution (*Top* and *Bottom*, green lines). The correct average peak position of the localizations is found from the radial histogram peak by subtracting 0.5*σ*^2^/*µ*^2^ [17,35] and superimposed onto the combined NPC localizations (see Section 2; *Top*, green). (**D**) The table shows *µ* ± *σ* calculated from A-C, number of NPCs analyzed for each condition, and *p*-values. (**E**) Mean SML radial distances from the center for three biological replicates. For one biological replicate (red symbol), the cells were treated with 2 µM of Nocodazole in addition to Ant-A ATP depletion (Supplementary Figure S6). Mean ± SD are plotted. (See Supplementary Figures S4–S6 for results and analyses of independent replicates).

We next calculated the mean radius of NPCs from the superimposed single-molecule localizations (Figure 3). After aligning the SMLs of the selected pores, the radial distance from center was measured by fitting the SML distributions with a Gaussian function, from which we calculated the mean (*µ*) and standard deviation (*σ*) (Figure 3A–C, top and bottom). The calculated mean NPC radius for (+)Gluc samples was 51 ± 12.9 nm, and for the ATP depleted samples, NaN_3_ and Ant-A, it was 51 ± 12.8 nm and 51 ± 13.9 nm, respectively (Figure 3A–D). The difference between the NPC radius in ATP depleted and (+)Gluc samples was not significant (Figure 3D), and this result was consistent across three biological replicates (Figure 3E, and Supplementary Figures S4–S6).

We repeated the ATP depletion experiment by pre-incubating cells for 4 h with Ant-A and treating with 2 µM of the microtubule (MT) polymerization inhibitor Nocodazole (Noco, Supplementary Figure S6). Nocodazole was used since MTs are known to interact with the NPC [49], so their depolymerization could affect the NPC radius. We saw changes in nuclear envelope morphology in cells treated with Ant-A + Noco compared to (−)Gluc samples or cells treated with Ant-A alone (Supplementary Figure S6A). We then calculated the pore radius distributions for (−)Gluc and Ant-A + Noco cells. The mean pore radius did not significantly change between (−)Gluc (51 ± 13.4 nm) and Ant-A + Noco (51 ± 13.5 nm) (Supplementary Figure S6D,F). These results show that depleting ATP, alone or combined with MT depolymerization, does not cause changes in the NPC radius in U2OS cells that can be detected by single-molecule localization microscopy.

### 3.3. NPC Size Is Resistant to Stretching and Shrinking of the Nuclear Envelope

**Figure 4:**
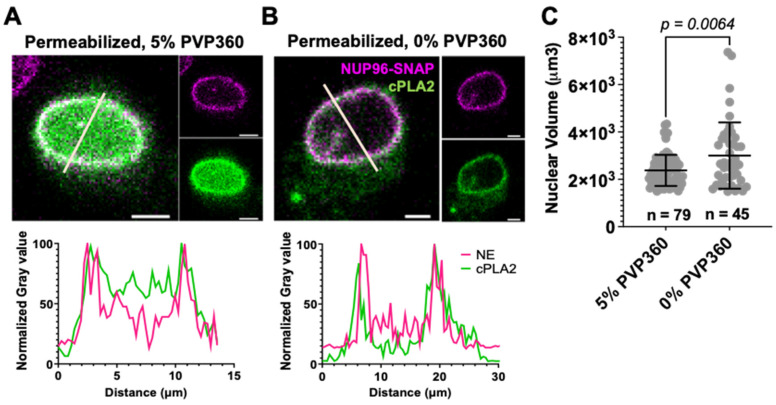
Osmotic swelling of nuclear membrane. (**A**,**B**) U2OS NUP96-SNAP cells were transfected with the membrane tension sensor, cPLA2-GFP (green). Twenty-four hours after transfection, cells were permeabilized for 5 min with 25 µg/mL digitonin in base medium in the presence of 5% Polyvinylpyrrolidone (PVP360) to prevent cell swelling. Scale bar 5 μm. (**A**,**B**). Permeabilization medium was removed, and cells were further incubated with 5% PVP360 for 5 min (**A**) or in 0% PVP360 for 15 min (**B**). Pre-permeabilized samples were fixed and stained with SNAP-AF647 dye (magenta). *Left*: Representative images of a middle section of the nuclear envelope (NE). *Right*: Images of individual cPLA2 and SNAP channels. Below each image are line histograms of the normalized intensities of cPLA2 (green) and SNAP (NE, magenta), corresponding to lines drawn across the nuclei on the confocal images. (**C**) Distributions of nuclear volumes (in µm^3^) of stretched (0% PVP360) and non-stretched (5% PVP360) nuclei. Shown are means ± SD, *n* = Number of nuclei. Statistical analysis was done using Welch’s *t*-test.

Changes in nuclear membrane tension have been reported to alter the NPC diameter [13,14]. We sought to osmotically swell the nucleus to determine whether stretching of the nuclear membrane would expand the NPCs. To monitor the NE tension, we transfected U2OS NUP96-SNAP cells with the membrane tension sensor, cytosolic phospholipase A_2_ fused to GFP (cPLA_2_-GFP). This sensor binds to stretched nuclear membranes in a calcium-dependent manner [29,30]. We then permeabilized the cells with digitonin in the presence or absence of 5% Polyvinylpyrrolidone (PVP360), a polymer that prevents cell swelling by maintaining osmotic balance. cPLA_2_-GFP is evenly distributed across the nucleus in non-swelled nuclear membranes of permeabilized cells in the presence of 5% PVP360 (Figure 4A). However, in cells with stretched nuclear membranes (0% PVP360), cPLA_2_-GFP localizes primarily to the nuclear envelope (Figure 4B), thus confirming the generation of tension in the NE. To independently verify swelling of the nucleus, we also measured the nuclear volume (Figure 4C). Cells incubated with 5% PVP360 had significantly smaller nuclear volume (2.4 ± 0.9 × 10^3^ µm^3^) than those incubated with 0% PVP360 (3.1 ± 1.5 × 10^3^ µm^3^), confirming nuclear membrane stretching.

**Figure 5:**
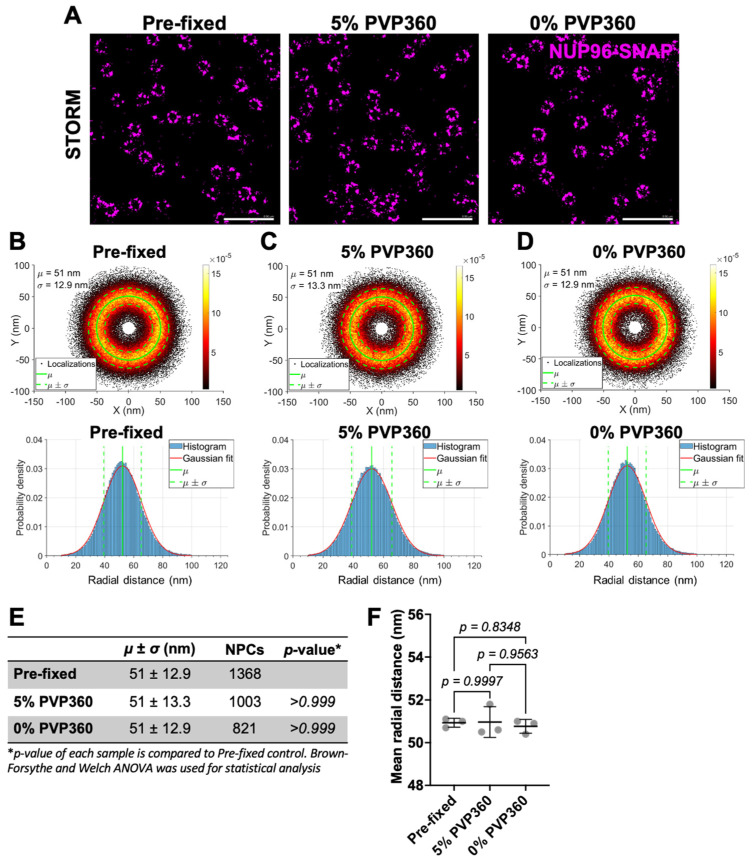
Stretching the nuclear envelope does not change the NPC radius. (**A**) STORM images of U2OS NUP96-SNAP cells either fixed before permeabilization (Pre-fixed) or pre-permeabilized with 25 µg/mL of digitonin and incubated under swelling (0% PVP360) or not swelling (5% PVP360) conditions, as in Figure 4. Cells fixed prior to permeabilization were used as a control. NUP96-SNAP was stained with the SNAP-AF647 dye. Shown are representative images at the bottom of the nuclear envelope. Scale bar: 0.5 µm. (**B**–**D**) Same as in Figure 3. *Top*: Scatter plots showing the radial distributions of single-molecule localization densities of superimposed NPCs selected from (**A**), aligned at their center of mass. Shown right is the pseudo color scale for the localization density. *Bottom*: SML radial distance histograms were fitted with a Gaussian function (red). The *µ* and *σ* were calculated from the fitted Gaussian distribution (*Top* and *Bottom*, green lines). (**E**) The table shows *µ* ± *σ* calculated on (**B**–**D**), number of NPCs analyzed for each condition and *p*-values. (**F**) Mean SML radial distances from the center for three biological replicates. Mean ± SD are plotted. (See Supplementary Figures S7 and S8 for analysis of independent replicates).

STORM imaging of stretched and unstretched nuclear membranes yielded the mean of NPC radius for permeabilized cells in the presence or absence of 5% PVP360 of 51 ± 13.3 nm and 51 ± 12.9 nm, respectively. In control experiments, the mean radius of samples fixed before permeabilization was about 51 ± 12.9 nm, all in excellent agreement with Figure 3 (Figure 5B–E). We found no significant change in the mean radial distance between each condition across three biological replicates (Figure 5F and Supplementary Figures S7 and S8). In summary, our results suggest that osmotically stressing the nuclear membrane does not cause detectable changes in the pore radius.

In addition to osmotically swelling the nucleus, we also tested the effect of shrinkage through exposure to hypertonic buffer [13,14]. We incubated U2OS NUP96-SNAP cells in 0 M or 2 M of sorbitol for 1 h, fixed and stained for NUP96-SNAP. As expected, the nuclear volume was significantly reduced in cells treated with 2 M of sorbitol compared to the control (Supplementary Figure S9A,B). Interestingly, the difference in the overall mean pore radius was highly significant (Supplementary Figure S9C–E). However, the difference was ~0.3 nm and, in addition, falls within the standard deviation, which suggests that the significant difference might be due to high sample size and does not necessarily reflect significant changes in the pore radius.

## 4. Discussion

The nuclear pores have been used as reference for SML imaging by using antibodies, direct tags like self-labeling SNAP or Halo tags that covalently react with organic dyes, or by transient interactions with labeled DNA strands to perform DNA-PAINT imaging [27,37,50]. DNA-PAINT affords better spatial resolution while yielding the NPC radius close to that measured by direct STORM of NUP96-SNAP using SNAP-AF647 dye [27,37]. Our STORM imaging of U2OS NUP96-SNAP cells yielded a mean NPC radius of ~51 nm. The spatial STORM resolution of AlexaFluor647-labeled NUP96-SNAP pores estimated by FRC, SML alignment, and Gaussian fitting was ~13 nm, which is consistent to the previously established spatial resolution of ~13.3 nm by the same group [27]. Within this limit, we did not detect significant changes in the mean NPC radius after ATP depletion or upon stretching/shrinking the nuclear envelope through osmotic stresses. In some cases, difference in the mean radius would appear significant, but the actual change in the radius was less than 1 nm, which is more than 10 times less than previous studies showing changes in NPC radius and would rather suggest sample-to-sample variation and/or a result of a very large SML sample size.

Our findings contrast with the cryo-ET study reporting contraction of yeast NPCs in the absence of ATP and under hypertonic conditions [13]. This discrepancy may be related to the differences in yeast and mammalian NPC composition. For example, *Saccharomyces cerevisiae* has 16 total copies of the Y-complex scaffold per NPC, compared to 32 copies in the human NPC [3,48,51,52]. Similarly, *Schizosaccharomyces pombe* has an asymmetric Y-complex distribution across the NPC, with more subunits in the nucleoplasmic ring (NR) than in the cytoplasmic ring (CR) [13,48]. ATP depletion in *S. pombe* resulted in a marked reduction of pore’s diameter by ~20 nm at the CR and inner ring (IR), while the NR diameter was reduced by only ~8 nm [13]. This result suggests that the double Y-complex at the NR in *S. pombe* cells may provide more rigidity compared to CR and IR. In addition, the Y-complex of *S. cerevisiae* lacks two NUPs, which are present in the human Y-complex [3,48,51,52]. These structural differences may allow the yeast NPC to respond more readily to energy depletion and mechanical stimuli than the human nuclear pore. Note that depletion of the members of Y-complex, NUP96 or NUP133, resulted in a loss of NR and CR spokes and increased the IR diameter in mammalian cells [11,15]. This result supports the role of Y-complex in regulating the rigidity and symmetry of the NPC, while the IR might be more flexible and prone to change its diameter than the CR and NR [11,15]. Future studies using endogenously tagged NUPs located in the central channel, such as NUP54, may enable detection of NPC remodeling by STORM. STORM Imaging of NUP54 will likely yield a smaller apparent pore radius, thereby providing a positive control for our ability to resolve changes in the NPC size.

In addition to energy depletion, changes in NE tension have also been shown to impact the NPC size and permeability [13,14,53,54]. Hypertonic conditions appear to shrink the nucleus and reduce the NPC diameter in yeast and mammalian cells [13,14]. Conversely, stretching the NE increases the NPC size, as determined by cryo-ET, and induces nuclear translocation of the mechanosensing YAP transcription factor [53,54]. However, such mechanical stress-induced changes in pore diameter were not detectable by our STORM imaging. One reason for the apparent discrepancy with our data may be the inferior spatial resolution of STORM imaging compared to cryo-ET and TEM used by others. However, these studies showed changes of about 20 nm in NPC diameter, which falls within the resolution limit of STORM. In addition, expansion microscopy [14], which achieves a similar spatial resolution to that of STORM, detected NPC shrinking in human cells by hypertonic shock. We also cannot rule out possible paraformaldehyde fixation artifacts masking the pore remodeling, but such prefixation has also been used for expansion microscopy [14]. In addition, labeling efficiency of SNAP with SNAP-AF647 dye and SNAP-tagging of NUP96 might affect the resolution limit or the NPC structure and dynamics, respectively. While the reasons for these apparent discrepancies are presently unclear, detection of morphological changes in NPC could be affected by the cell type, method used for sample preparation, and/or imaging modality.

Our results indicate a surprising resistance of NPCs of human U2OS cells to mechanical stimuli and the lack of reliance on active ATP energy-dependent processes for maintaining the pore architecture. Since cryo-ET studies have revealed a marked reduction in pore diameter in isolated nuclei vs. intact cells [5,10,11,24], our future STORM experiments will compare the NPC diameters in intact U2OS cells and isolated nuclei. In addition, we will employ a DNA-PAINT approach to achieve a higher photon budget and better localization precision [37]. Once our ability to detect NPC remodeling is validated, we will employ 2-color STORM to assess the effects of HIV-1 docking on the shape and diameter of nuclear pores.

## Data Availability

Original STORM data related to this research are available from the corresponding author upon request.

## Supplementary Materials

The following supporting information can be downloaded at: https://www.mdpi.com/article/10.3390/v18020167/s1. Figure S1: HIV-1 infection of U2OS endogenously expressing NUP96-SNAP. Figure S2: ATP depletion in U2OS NUP96-SNAP cells, second biological replicate. Figure S3: Spatial resolution of (+)Gluc treated cells. Figure S4: Effects of ATP depletion on the NPC size distribution (second replicate). Figure S5: Effect of ATP depletion with NaN_3_ on the NPC size, third replicate. Figure S6: Effects of ATP depletion with Antimycin A and microtubule depolymerization with Nocodazole on NPC size. Figure S7: Effects on nuclear envelope swelling on NPC size (second biological replicate). Figure S8: Effects on nuclear envelope swelling on NPC size (third biological replicate). Figure S9: Shrinkage of the nuclear envelope with hypertonic medium does not change the NPC radius.
