# Supplementary material for: Super-Resolution Imaging of Nuclear Pore Responses to Mechanical Stress and Energy Depletion
Source: Viruses. 2026 Jan 27;18(2):167. doi: 10.3390/v18020167 (PMC12945098; doi:10.3390/v18020167)
Supplement: Supplementary file 1 [file viruses-18-00167-s001.zip › viruses-4039847-supplementary.pdf]

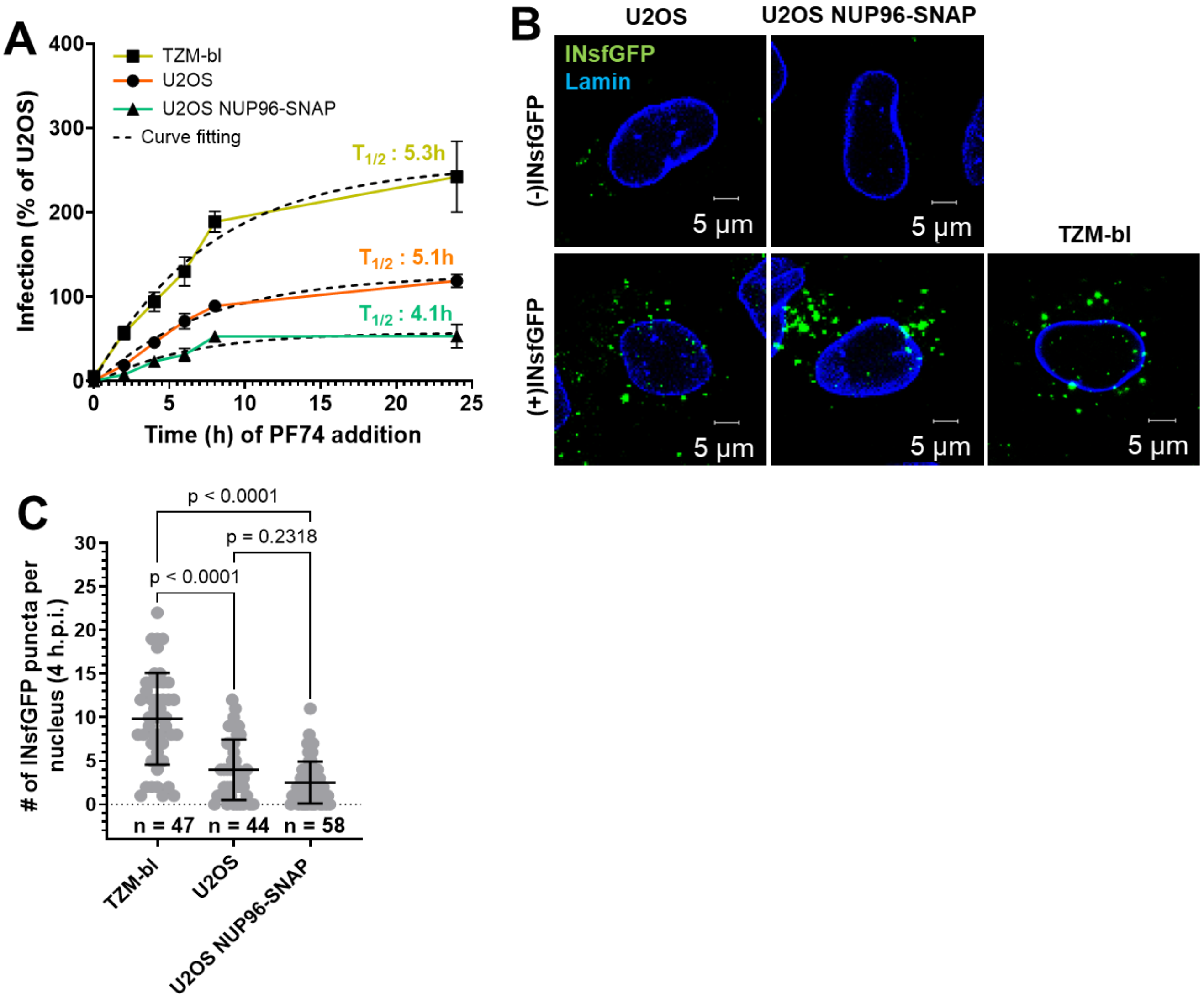

Suppl. Figure S1: HIV-1 infection of U2OS endogenously expressing NUP96-SNAP. (A) TZM-bl, U2OS and U2OS NUP96-SNAP cells were infected with NL4-3 Luciferase HIV-1 virus pseudotyped with VSV-G. The kinetics of HIV-1 nuclear import was assessed by adding 2 µM PF74 (or DMSO) at indicated time points post infection (p.i.). Luciferases activity was measured 48 h.p.i. and normalized to signal registered from DMSO treated parental U2OS cells. Half-times for HIV-1 nuclear import ($T_{1/2}$) were determined by curve fitting with an exponential function (dashed lines). Data is plotted as Mean ± Standard Deviation (SD). (B) Confocal images of TZM-bl, U2OS and U2OS NUP96-SNAP cells infected or non-infected with equal amounts of NL4-3 HIV-1 pseudotyped with VSV-G and fluorescently labeled with Integrase-superfolder GFP (INsfGFP, green). Cells were fixed and immunostained for Lamin (blue) at 4 h.p.i. Shown are maximum intensity projection images of 2-3 middle optical sections of the cell. (C) Number of INsfGFP virus particles per nucleus from images shown in panel B. TZM-bl cells nuclear import experiments were performed under conditions identical to those used for U2OS cells. Shown are mean ± SD, n = number of nuclei analyzed. Statistical analysis was done with Kruskal-Wallis test.

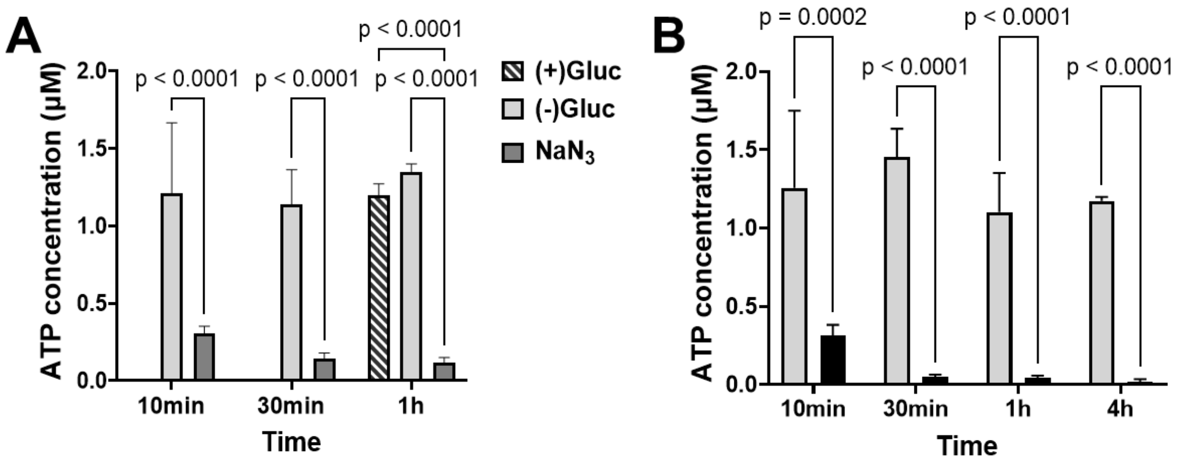

Suppl. Figure S2: ATP depletion in U2OS NUP96-SNAP cells, second biological replicate. Same as Figure 1. (A) Cells were incubated in (+)Gluc, (-)Gluc or in NaN_3 ATP depletion medium (10 mM NaN_3 and 6 mM 2-deoxy-D-glucose) for varied times. The cellular ATP concentration (in µM) was measured in triplicate samples using a kit and plotted as mean ± SD. (B) Same as in A but cells were depleted of ATP with 10 µM of Antimycin A and 20 mM 2-deoxy-D-glucose (Ant-A) for different times. ATP concentration in cells was measured and plotted as mean ± SD of 3 technical replicates. Statistical analysis was done with 2-way ANOVA.

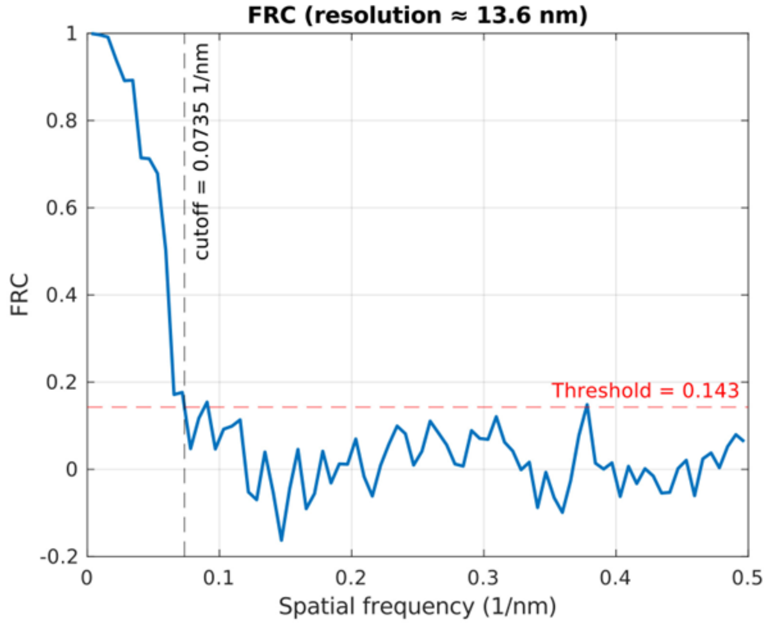

Suppl. Figure S3: Spatial resolution of (+)Gluc treated cells. Fourier Ring Correlation (FRC) of (+)Gluc control sample. FRC was calculated by dividing the dataset into two halves and each half was registered, applied eight-fold symmetry to each particle and computed the FRC curve. The resolution of the reconstruction was determined by applying a resolution threshold to the FRC curve. The estimated resolution is ~13.6 nm.

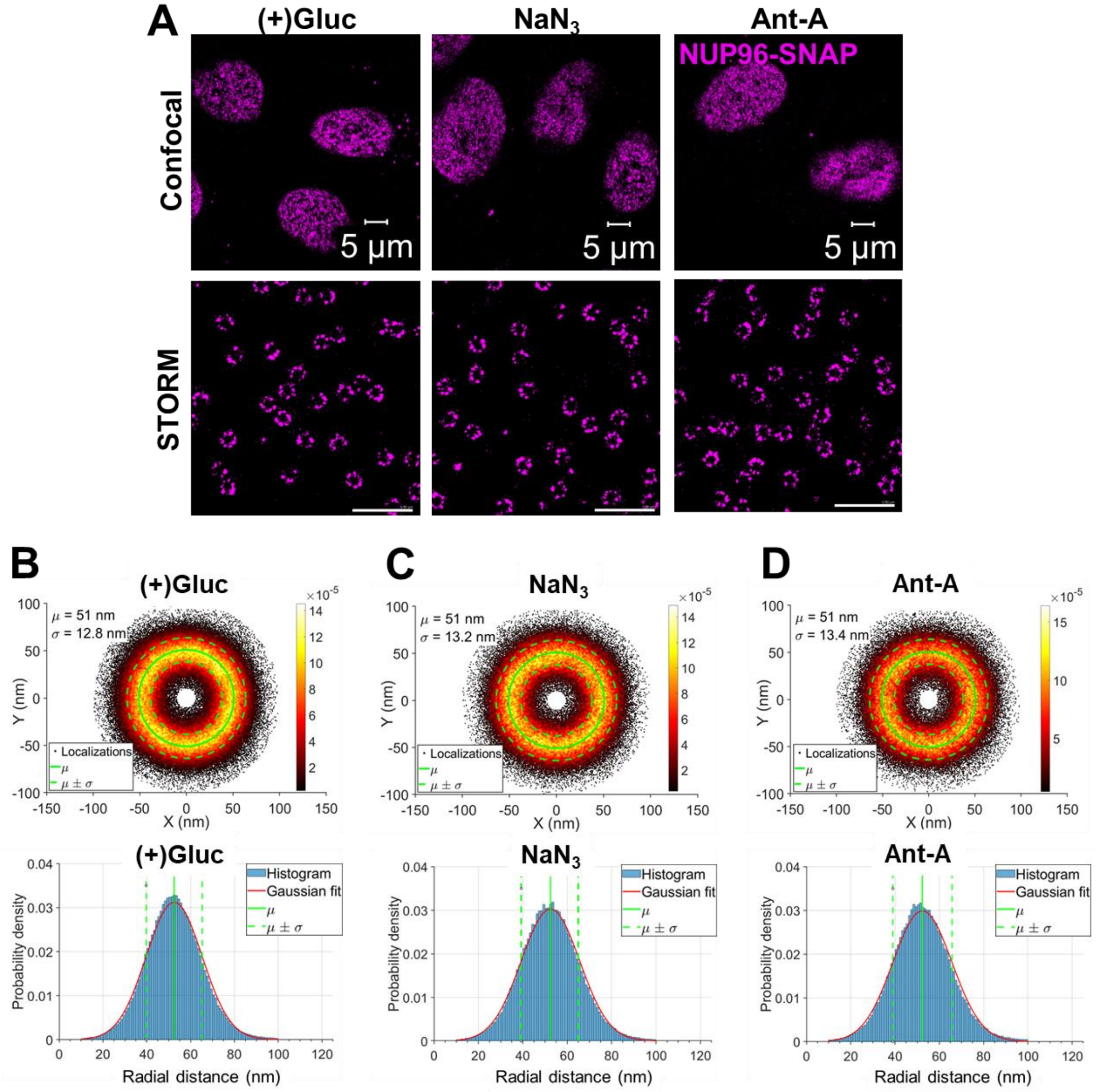

E

	$\mu \pm \sigma$ (nm)	NPCs	*p*-value*
**(+)Gluc**	51 ± 12.8	1129	
**NaN_3**	51 ± 13.2	1189	*>0.999*
**Ant-A**	51 ± 13.4	943	*>0.999*

**p-value of each sample is compared to (+)Gluc control. Brown-Forsythe and Welch ANOVA was used for statistical analysis*

Suppl. Figure S4: Effects of ATP depletion on the NPC size distribution (second replicate). (A) _Top_: Confocal images of U2OS NUP96-SNAP cells incubated in complete medium ((+)Gluc) and ATP-depletion media, NaN_3 or Ant-A, for 2 h. Cells were fixed, permeabilized and NUP96-SNAP was stained with the SNAP-AF647 dye. Shown are representative images of confocal slices at the bottom of the nuclear envelope. _Bottom_: STORM images of NPCs at the bottom of the nuclear envelope. Scale bar: 0.5 µm. (B-D) Same as in Figure 3. _Top:_ Scatter plots showing the localizations of super imposed NPCs selected on A, after aligning the center of mass, transformed the coordinates into polar form and super imposed to form a final averaged NPC. Shown right is the pseudo color scale for the localization density. _Bottom_: SML radial distance histograms were fitted with a Gaussian function (red). The μ and σ were calculated from the fitted Gaussian distribution (_Top_ and _Bottom,_ green lines). (E) Table shows $\mu \pm \sigma$ calculated on B-D, number of NPCs analyzed for each condition and *p*-values.

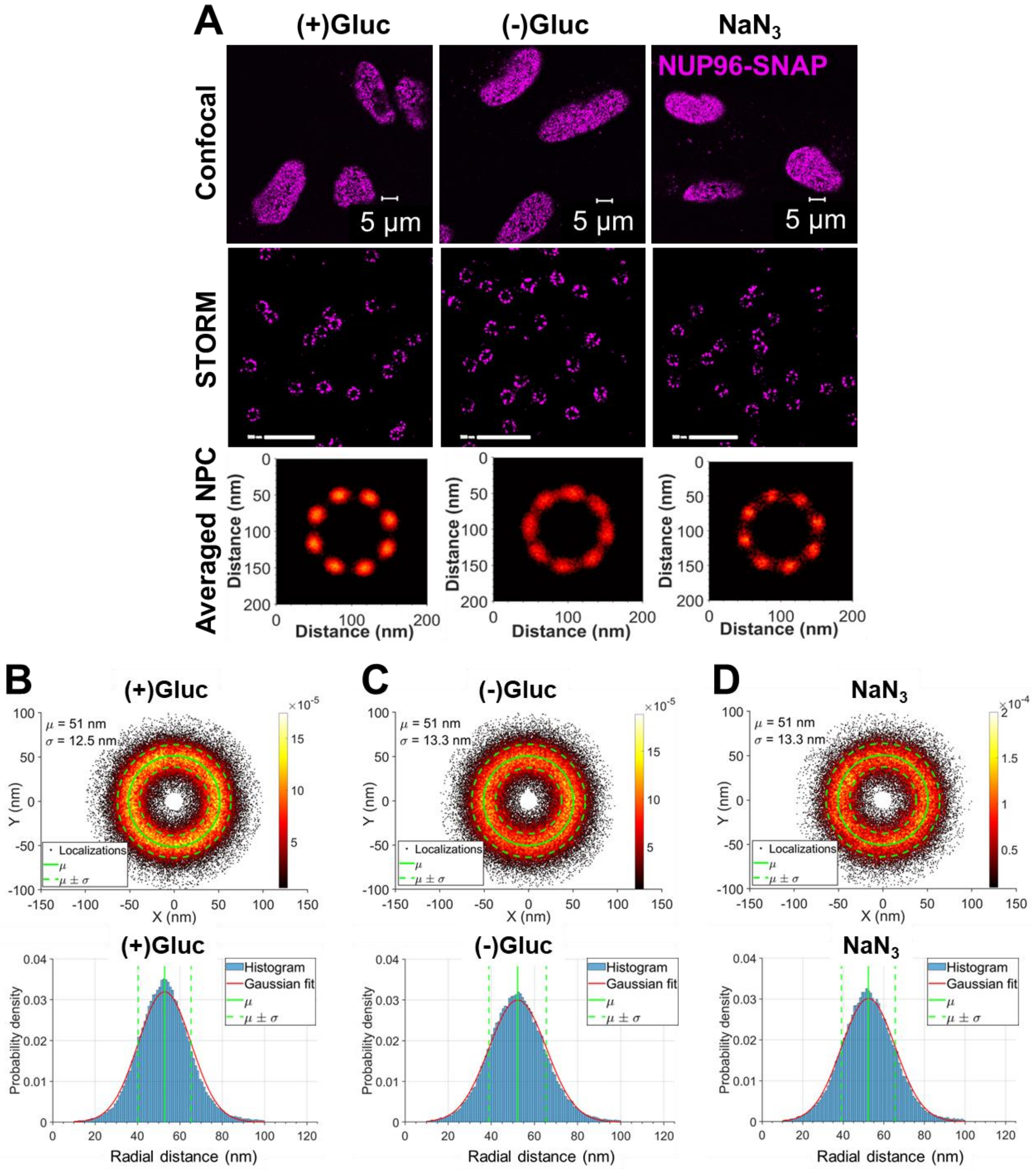

E

	$\mu \pm \sigma$ (nm)	NPCs	*p*-value*
**(+)Gluc**	51. ± 12.5	1157	
**(-)Gluc**	51 ± 13.3	1144	*>0.999*
**NaN_3**	51 ± 13.3	826	*>0.999*

**p-value of each sample is compared to (+)Gluc control. Brown-Forsythe and Welch ANOVA was used for statistical analysis*

Suppl. Figure S5: Effect of ATP depletion with NaN_3 on the NPC size, third replicate. (A) *Top*: Confocal images of U2OS NUP96-SNAP cells incubated in (+)Gluc, (-)Gluc buffer, and ATP depletion medium with NaN_3 for 1h. Cells were fixed, permeabilized and stained with the SNAP-AF647 dye. Shown are representative images of confocal slices at the bottom of the nuclear envelope. *Middle*: STORM images of NPCs at the bottom of the nuclear envelope. Scale bar: 0.5 µm. *Bottom*: Averaged NPC images from STORM localizations. (B-D) Same as in Figure 3. *Top:* Scatter plots showing the localizations of super imposed NPCs selected on A, after aligning the center of mass, transformed the coordinates into polar form and super imposed to form a final averaged NPC. Shown right is the pseudo color scale for the localization density. *Bottom*: SML radial distance histograms with a fitted Gaussian function (red). The μ and σ were calculated from the fitted Gaussian distribution (*Top* and *Bottom,* green lines). (E) Table shows $\mu \pm \sigma$ calculated on B-D, number of NPCs analyzed for each condition and *p*-values.

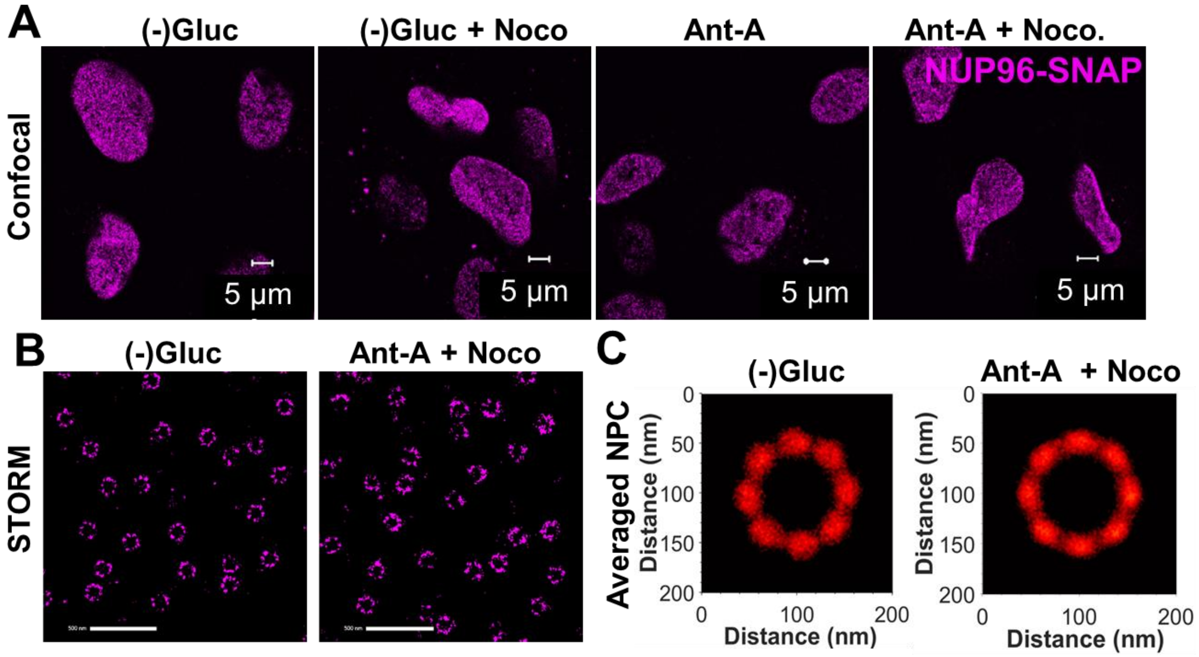

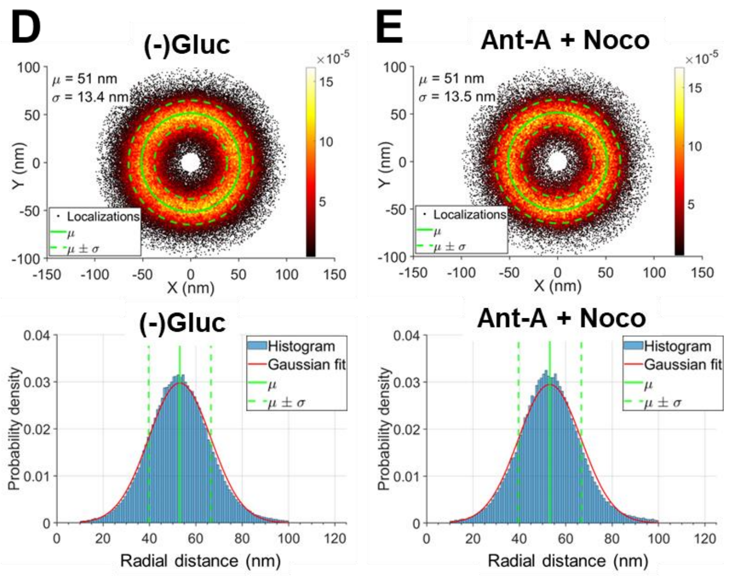

F

	$\mu \pm \sigma$ (nm)	NPCs	*p*-value*
**(-)Gluc**	51 ± 13.4	1406	
**Ant-A + Noco**	51 ± 13.5	685	*>0.999*

**p-value is compared to (-)Gluc control. Welch's t-test was used for statistical analysis*

Suppl. Figure S6: Effects of ATP depletion with Antimycin A and microtubule depolymerization with Nocodazole on NPC size. (A) Confocal images of ATP depleted cells with or without Nocodazole. Cells were incubated in glucose-free medium ((-)Gluc) or in ATP depletion medium (10 µM of Antimycin A and 20 mM 2-deoxy-D-glucose, dubbed Ant-A) with or without the microtubule polymerization inhibitor Nocodazole (2 µM, Noco). Cells were fixed, permeabilized and stained for NUP96-SNAP with SNAP-AF647 dye. Shown are representative images of the bottom of the nuclear envelope after 4 h of treatment. (B) STORM images of NPCs in (-)Gluc and in ATP depleted with Ant-A + Noco after a 4 h treatment. Scale bar: 0.5 µm. (C) Images of averaged NPCs from STORM data in B. (D-E) Same as in Figure 3. ***Top:*** Scatter plots showing the localizations of super imposed NPCs selected on B, after aligning the center of mass, transformed the coordinates into polar form and super imposed to form a final averaged NPC. Shown right is the pseudo color scale for the localization density. ***Bottom***: SML radial distance histograms with a fitted Gaussian function (red). The μ and σ were calculated from the fitted Gaussian distribution (***Top*** and ***Bottom***, green lines). (F) Table shows $\mu \pm \sigma$ calculated on D-E, number of NPCs analyzed for each condition and *p*-values.

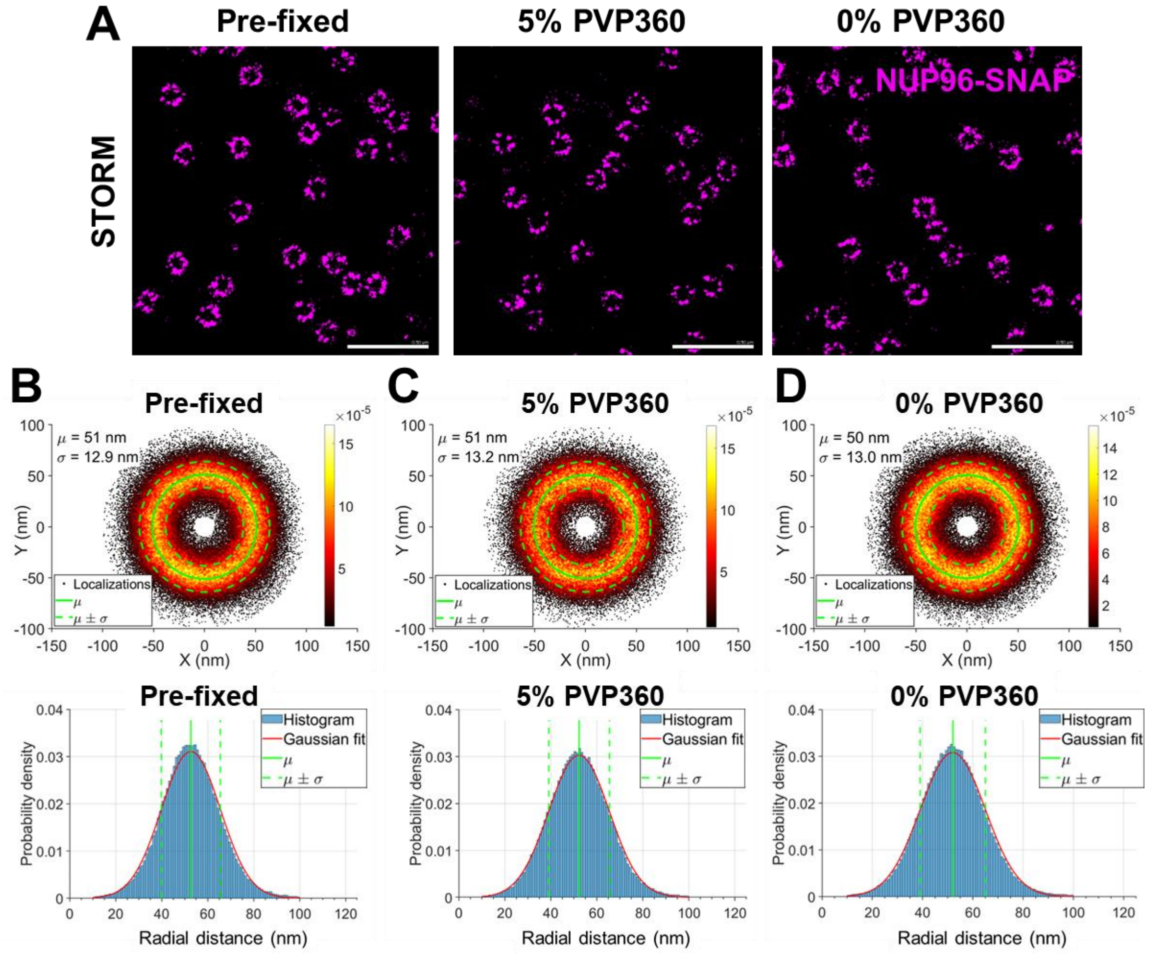

E

	$\mu \pm \sigma$ (nm)	NPCs	*p*-value*
**Pre-fixed**	51 ± 12.9	1221	
**5% PVP360**	51 ± 13.2	1064	*>0.999*
**0% PVP360**	50 ± 13.0	1252	*>0.999*

**p-value of each sample is compared to Pre-fixed control. Brown-Forsythe and Welch ANOVA was used for statistical analysis*

Suppl. Figure S7: Effects on nuclear envelope swelling on NPC size (second biological replicate). (A) STORM images of U2OS NUP96-SNAP cells pre-fixed or under swelling (0% PVP360) or non-swelling (5% PVP360) conditions, as in Figure 4. NUP96-SNAP was stained with the SNAP-AF647 dye. Shown are representative images at the bottom of the nuclear envelope. Scale bar: 0.5 µm. (B-D) Same as in Figure 3. *Top:* Scatter plots showing the localizations of super imposed NPCs selected on A, after aligning the center of mass, transformed the coordinates into polar form and super imposed to form a final averaged NPC. Shown right is the pseudo color scale for the localization density. *Bottom*: SML radial distance histograms with a fitted Gaussian function (red). The μ and σ were calculated from the fitted Gaussian distribution (*Top* and *Bottom,* green lines). (E) The table shows $\mu \pm \sigma$ calculated on B-D, number of NPCs analyzed for each condition and *p*-values.

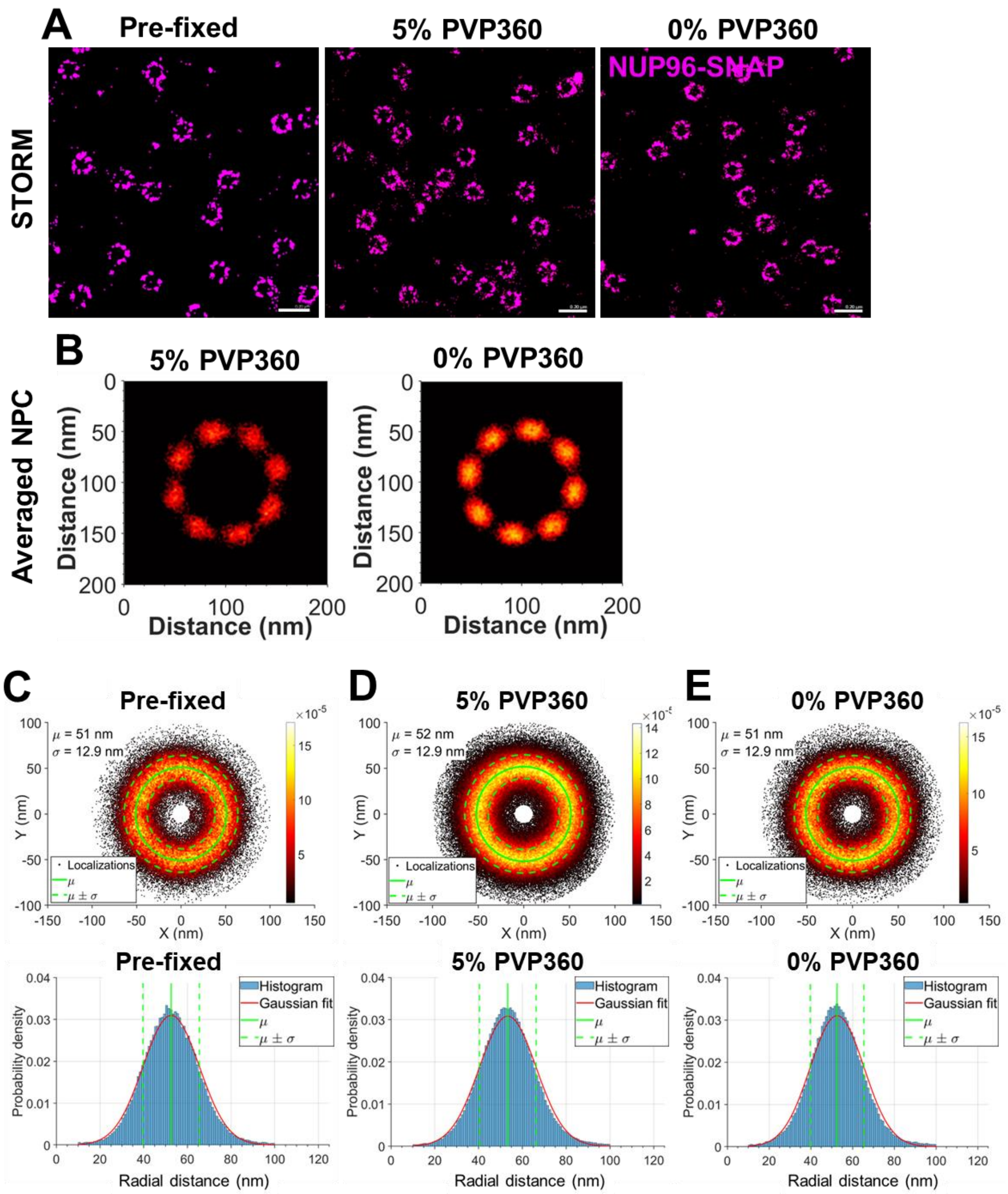

F

	$\mu \pm \sigma$ (nm)	NPCs	*p*-value*
**Pre-fixed**	51 ± 12.9	1049	
**5% PVP360**	52 ± 12.9	2470	*>0.999*
**0% PVP360**	51 ± 12.9	1201	*>0.999*

**p-value of each sample is compared to Pre-fixed control. Brown-Forsythe and Welch ANOVA was used for statistical analysis*

Suppl. Figure S8: Effects on nuclear envelope swelling on NPC size (third biological replicate). (A) STORM images of NPCs in swelled and control nuclear membranes. U2OS NUP96-SNAP cells were pre-fixed or under swelling (0% PVP360) or not swelling (5% PVP360) conditions, as in Figure 4. NUP96-SNAP was stained with the SNAP-AF647 dye. Shown are images of pores at the bottom of the nuclear envelope. Scale bar: 0.2 µm. (B) Averaged NPC from STORM data for 0% PVP360 and 5% PVP360. (C-E) Same as in Figure 3. *Top:* Scatter plots showing the localizations of super imposed NPCs selected on A, after aligning the center of mass, transformed the coordinates into polar form and super imposed to form a final averaged NPC. Shown right is the pseudo color scale for the localization density. *Bottom*: SML radial distance histograms with a fitted Gaussian function (red). The μ and σ were calculated from the fitted Gaussian distribution (*Top* and *Bottom,* green lines). (F) Table shows $\mu \pm \sigma$ calculated on C-E, number of NPCs analyzed for each condition and *p*-values.

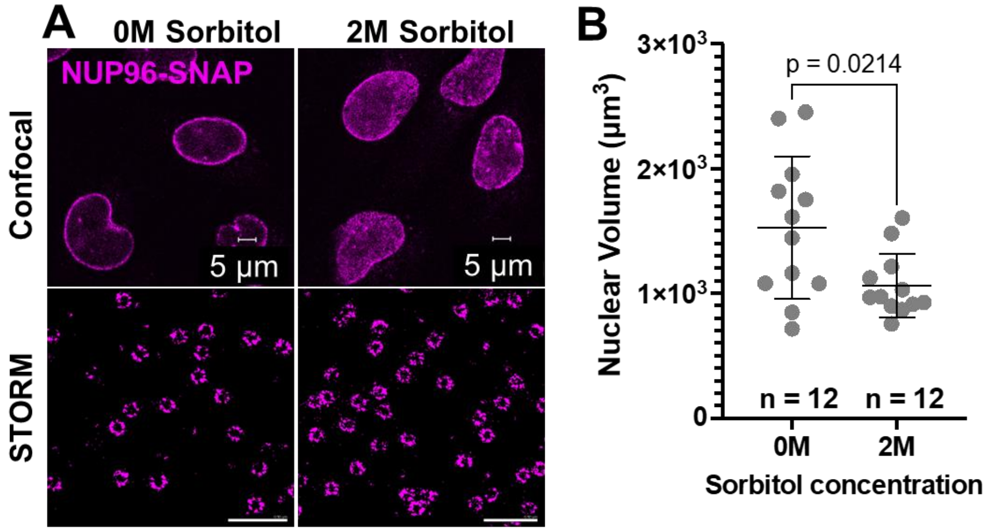

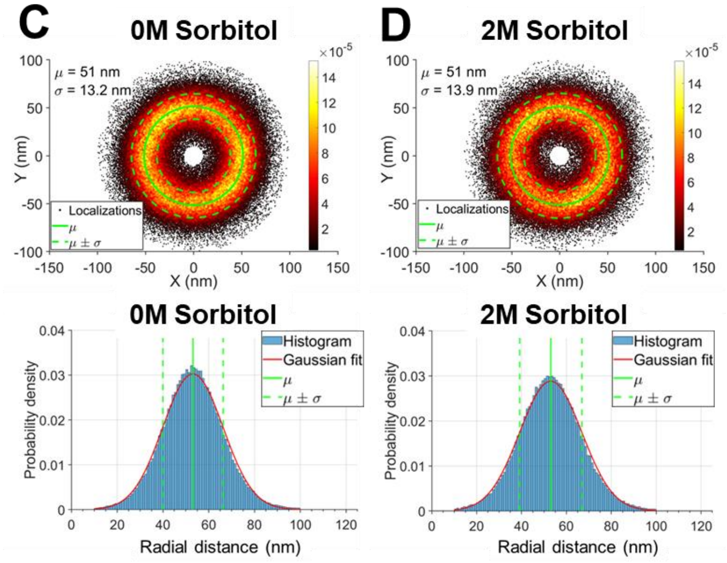

E

	**μ ± σ (nm)**	**NPCs**	***p*-values***
**0M Sorbitol**	51 ± 13.2	1462	
**2M Sorbitol**	51 ± 13.9	934	*<0.0001*

**p-value is compared to 0M sorbitol control. Welch's t-test was used for statistical analysis.*

Suppl. Figure S9: Shrinkage of the nuclear envelope with hypertonic medium does not change the NPC radius. (A) Confocal (top) and STORM images (bottom) of cells treated with or without 2M of sorbitol for 1h. Confocal images are representative of middle section of the NE. Scale bar for STORM images: 0.5 µm. (B) Volume (in μm^3) of nucleus from cells treated with 2M sorbitol and mock-treated (from panel A). Means ± SD are shown; Welch's t-test was used for statistical analysis, n = number of nuclei. (C-D) Same as in Figure 3. *Top:* Scatter plots showing the localizations of super imposed NPCs selected on A, after aligning the center of mass, transformed the coordinates into polar form and super imposed to form a final averaged NPC. Shown right is the pseudo color scale for the localization density. *Bottom*: SML radial distance histograms with a fitted Gaussian function (red). The μ and σ were calculated from the fitted Gaussian distribution (*Top* and *Bottom,* green lines). (E) Table shows $\mu \pm \sigma$ calculated on C-D, number of NPCs analyzed for each condition and *p*-values.
